# Supplementary material for: Sloth metabolism may make survival untenable under climate change scenarios
Source: PeerJ. 2024 Sep 27;12:e18168. doi: 10.7717/peerj.18168 (PMC11441404; doi:10.7717/peerj.18168)
Supplement: Supplemental Information 1 [file peerj-12-18168-s001.docx]

**Table S1.** Origin location, altitude, and climate information for the 12 *C. hoffmanni* sloths used for metabolic testing.

| **Sloth** | **Altitude category** | **Origin location** | **Origin altitude (meters above sea level)** | **Annual high temperature** ºC | **Annual low temperature** ºC |
| --- | --- | --- | --- | --- | --- |
| 1 | lowland | Las Horquetas, Sarapiquí | 68 | 30.22 | 24.42 |
| 2 | lowland | Sixaola, Talamanca | 10 | 31.73 | 24.84 |
| 3 | lowland | Penshurt, Limon | 5 | 31.15 | 26.03 |
| 4 | highland | Monteverde, Puntarenas | 1330 | 25.43 | 18.56 |
| 5 | lowland | Penshurt, Limon | 5 | 31.15 | 26.03 |
| 6 | lowland | Barrio la colina, Limon | 3 | 31.46 | 24.61 |
| 7 | highland | Escazu Park, San Jose | 1101 | 24.35 | 15.98 |
| 8 | lowland | Valle le Estrella, Limon | 18 | 30.01 | 25.35 |
| 9 | lowland | Sixaola, Talamanca | 10 | 31.73 | 24.84 |
| 10 | highland | Cantón de Zarcero, Alajuela | 1769 | 24.71 | 16.73 |
| 11 | highland | Curridabat, San Jose | 1208 | 24.78 | 15.9 |
| 12 | lowland | Cahuita, Limon | 2 | 32.15 | 26.03 |
