# Supplementary material for: Sloth metabolism may make survival untenable under climate change scenarios
Source: PeerJ. 2024 Sep 27;12:e18168. doi: 10.7717/peerj.18168 (PMC11441404; doi:10.7717/peerj.18168)
Supplement: Supplemental Information 2 [file peerj-12-18168-s002.docx]

| **Sloth** | **Altitude** | **Number of measurements** | **Sex** | **Mass (Kg)** | **Measured RMR** | | | | **Predicted RMR** | | | **T_a_ range (°C)** | **T_b_ range (°C)** | **RMR range (kJ/day)** |
| --- | --- | --- | --- | --- | --- | --- | --- | --- | --- | --- | --- | --- | --- | --- |
|  |  |  |  |  | **(ml O_2_/g/h)** | **(Kcal/day)** | **(kJ/day)** | **(kJ/Kg/day)** | | **Kleiber prediction**  **(kcal/day)** | **White and Seymour prediction (kcal/day)** |  |  |  |
| 1 | lowland | 1 | Male | 4.25 | 0.21 | 104.71 | 420.94 | 99.04 | | 207.20 | 146.76 | 29 | 34.6 | - |
| 2 | lowland | 1 | Male | 6.5 | 0.16 | 122.31 | 491.68 | 75.64 | | 284.96 | 195.93 | 29 | - | - |
| 3 | lowland | 2 | Male | 5.5 | 0.14 | 90.62 | 364.29 | 66.23 | | 251.40 | 174.89 | 33-35 | 34 | 363-365 |
| 4 | highland | 13 | Male | 4.6 | 0.21 | 113.25 | 455.26 | 98.97 | | 219.87 | 154.88 | 17-33 | 34.9-35.6 | 326-701 |
| 5 | lowland | 11 | Female | 6.9 | 0.26 | 214.79 | 863.47 | 125.14 | | 298.01 | 204.05 | 25-33 | - | 730-963 |
| 6 | lowland | 16 | Female | 5.1 | 0.22 | 134.26 | 539.73 | 105.83 | | 237.56 | 166.14 | 18-34 | 31.2-34.7 | 361-772 |
| 7 | highland | 11 | Male | 5.6 | 0.24 | 159.83 | 642.51 | 114.73 | | 254.82 | 177.05 | 16-34 | 33.1-35.7 | 454-845 |
| 8 | lowland | 7 | Male | 4.2 | 0.24 | 121.19 | 487.19 | 116.00 | | 205.37 | 145.59 | 18-29 | - | 360-598 |
| 9 | lowland | 10 | Female | 5.65 | 0.17 | 114.56 | 460.53 | 81.51 | | 256.53 | 178.12 | 18-34 | 31.9-33.7 | 360-566 |
| 10 | highland | 16 | Female | 5.4 | 0.32 | 210.16 | 844.83 | 156.45 | | 247.97 | 172.72 | 18-34 | 34.0-37.0 | 549-1280 |
| 11 | highland | 12 | Male | 5.05 | 0.26 | 155.05 | 623.28 | 123.42 | | 235.81 | 165.03 | 19-34 | 33.1-34.8 | 357-718 |
| 12 | lowland | 7 | Male | 4.7 | 0.32 | 181.11 | 728.06 | 154.91 | | 223.45 | 157.16 | 17-34 | 30.1-33.9 | 599-860 |
| **Mean** | | | | **5.29** | **0.23** | **143.49** | **576.81** | **109.82** | |  |  |  |  |  |

**Table S2.** Altitude origin, body mass, resting metabolic rate (RMR), allometric predictions and body temperature (Tb) data for 12 *C. hoffmanni* sloths at different ambient temperatures (Ta).
