# Supplementary material for: Sloth metabolism may make survival untenable under climate change scenarios
Source: PeerJ. 2024 Sep 27;12:e18168. doi: 10.7717/peerj.18168 (PMC11441404; doi:10.7717/peerj.18168)
Supplement: Supplemental Information 3 — The confidence intervals (CI) were calculated using a bootstrap approach adjusted for sample size, ensuring accurate representation of variability. [file peerj-12-18168-s003.docx]

**Table S3. RMR projections for high- and low-altitude sloths under different climate change scenarios.** The confidence intervals (CI) were calculated using a bootstrap approach adjusted for sample size, ensuring accurate representation of variability.

| **Climate change scenario (°C)** | **Mean RMR (kJ/kg/day)** | **95% CI Lower** | **95% CI Upper** | **CI Width** |
| --- | --- | --- | --- | --- |
| **High-altitude sloths** | | | | |
| -5 | 98.467 | 94.577 | 102.357 | 7.780 |
| -4 | 102.954 | 99.195 | 106.712 | 7.517 |
| -3 | 108.315 | 104.694 | 111.936 | 7.242 |
| -2 | 114.380 | 110.902 | 117.857 | 6.955 |
| -1 | 122.060 | 118.644 | 125.475 | 6.831 |
| 0 | 121.615 | 118.281 | 124.951 | 6.670 |
| +1 | 135.006 | 131.515 | 138.496 | 6.981 |
| +2 | 139.777 | 136.070 | 143.484 | 7.414 |
| +3 | 144.401 | 140.532 | 148.270 | 7.738 |
| **Low-altitude sloths** | | | | |
| -5 | 95.132 | 95.079 | 95.184 | 0.105 |
| -4 | 99.900 | 99.829 | 99.971 | 0.142 |
| -3 | 105.342 | 105.258 | 105.425 | 0.167 |
| -2 | 110.384 | 110.300 | 110.467 | 0.167 |
| -1 | 116.135 | 116.073 | 116.197 | 0.124 |
| 0 | 121.615 | 121.574 | 121.656 | 0.082 |
| +1 | 126.467 | 126.445 | 126.578 | 0.133 |
| +2 | 129.516 | 129.501 | 129.595 | 0.094 |
| +3 | 127.682 | 127.665 | 127.774 | 0.109 |
