# Supplementary material for: Sloth metabolism may make survival untenable under climate change scenarios
Source: PeerJ. 2024 Sep 27;12:e18168. doi: 10.7717/peerj.18168 (PMC11441404; doi:10.7717/peerj.18168)
Supplement: Supplemental Information 4 [file peerj-12-18168-s004.pdf]

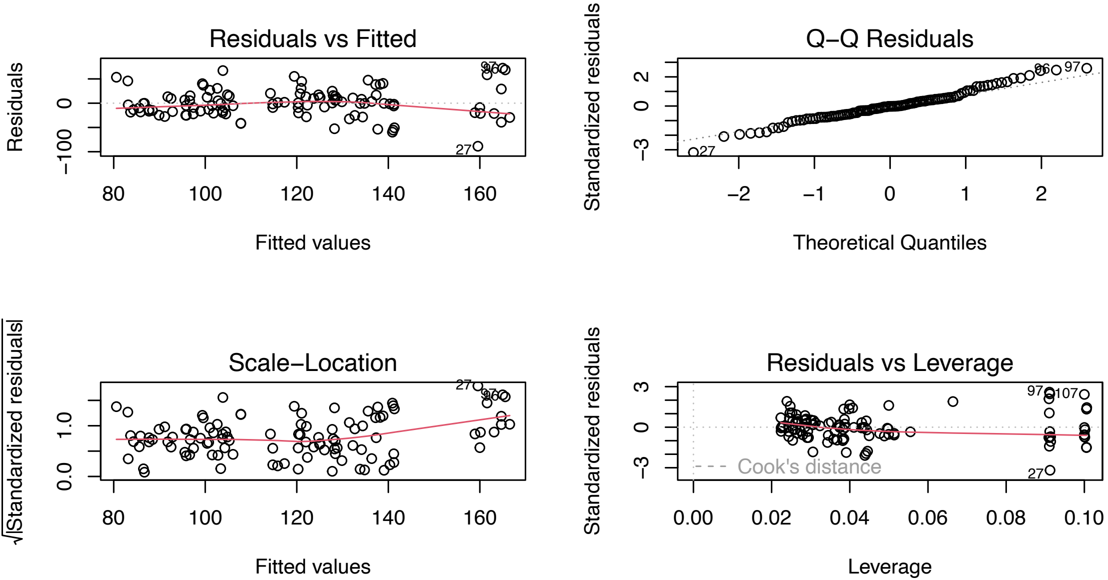
Residuals vs Fitted
Residuals
Fitted values
97
96
27
Q−Q Residuals
Standardized residuals
Theoretical Quantiles
96
97
27
Scale−Location
√|Standardized residuals|
Fitted values
27
97
96
Residuals vs Leverage
Standardized residuals
Leverage
97
107
27
Cook's distance
