# Supplementary figures and images for: Sloth metabolism may make survival untenable under climate change scenarios
Source: PeerJ. 2024 Sep 27;12:e18168. doi: 10.7717/peerj.18168 (PMC11441404; doi:10.7717/peerj.18168)

Low-altitude

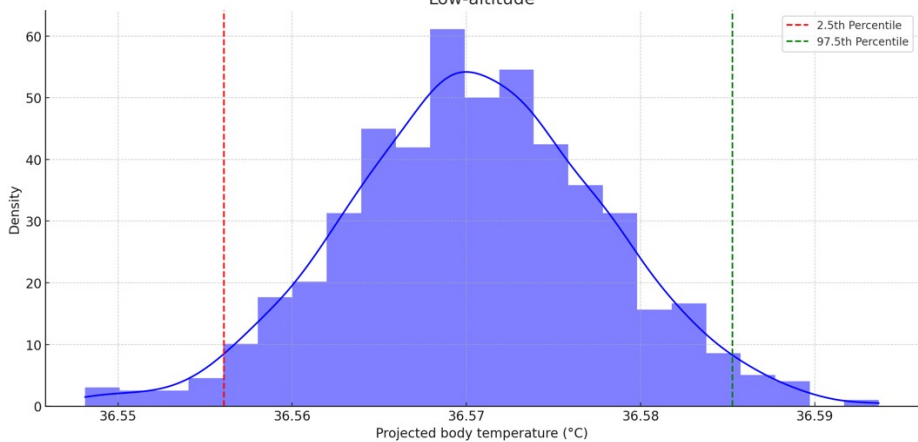

High-altitude

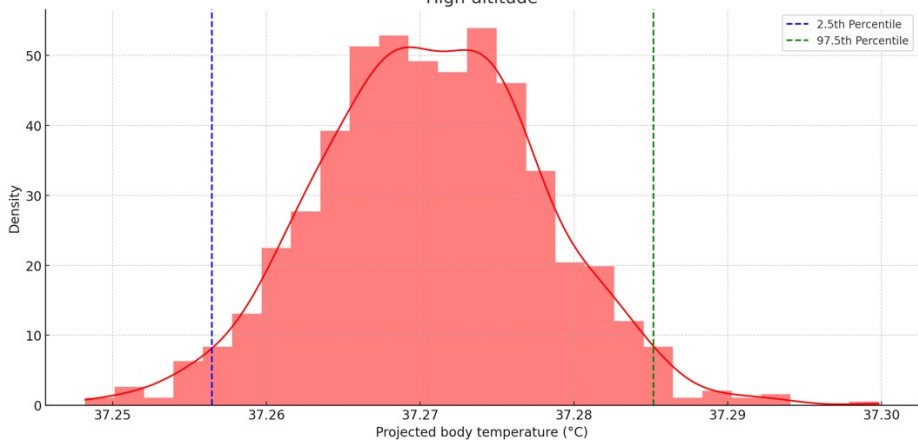

Supplement: Supplemental Information 5 — Predictions based on a 2 °C increase in ambient temperature. The top plot shows the distribution of projected body temperatures for low-altitude sloths, while the bottom plot shows the distribution for high-altitude sloths. The red dashed lines indicate the 2.5th percentiles, and the green dashed lines indicate the 97.5th percentiles of the projected temperatures, illustrating the range of uncertainty around the predictions. The density plots are derived from 1,000 bootstrap simulations to account for variability and uncertainty in the model predictions. [file peerj-12-18168-s005.pdf]

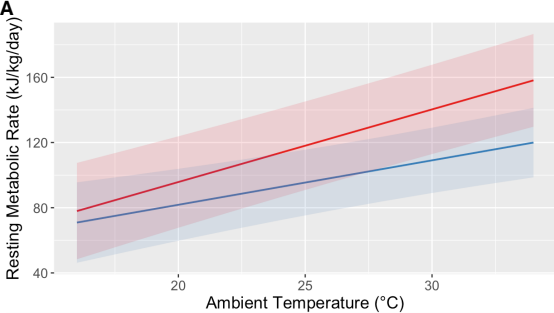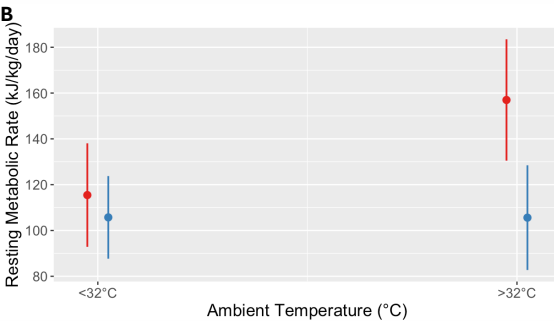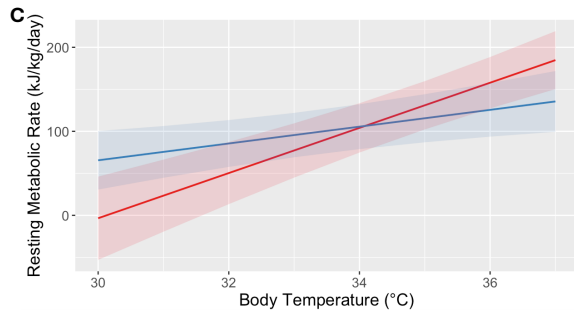

Supplement: Supplemental Information 6 — (A) Derived from LMM1, depicting ambient temperature on a continuous scale (the shaded areas represent 95% confidence intervals). (B) Derived from LMM2, depicting ambient temperature on a categorical scale. (C) The regression relationship between body temperature and RMR in high (red) and low (blue) altitude sloths, representing the interaction between altitude and body temperature on RMR (the shaded areas represent 95% confidence intervals). [file peerj-12-18168-s006.pdf]

(Intercept)

Vinnie

Oprah

Chow

Mandy

Mateo

Naima

David

David

Poko

Harpo

-40

-20

0

20

40

60

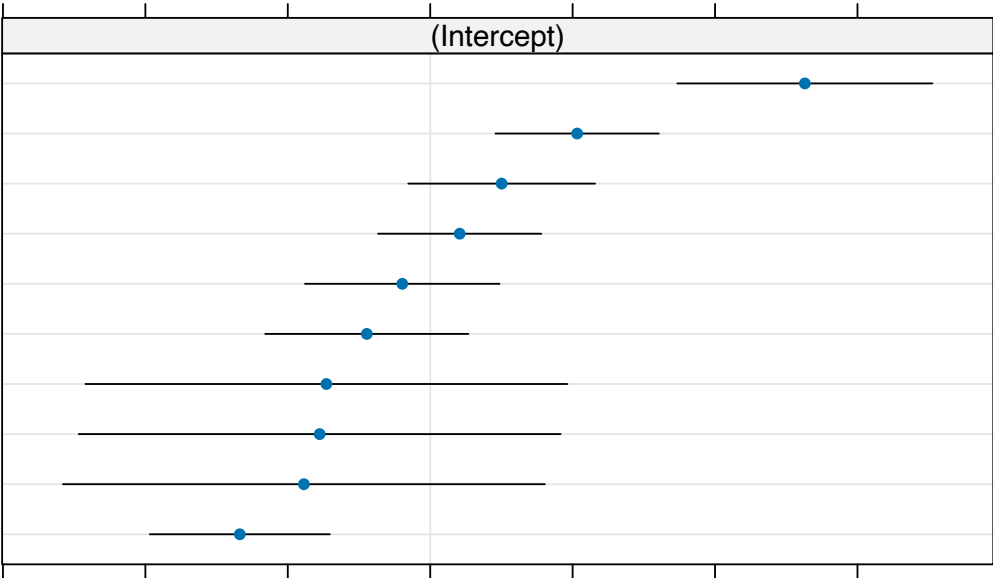

Supplement: Supplemental Information 7 — The intercept represents the population mean RMR, with blue dots representing the mean distance from the population mean for each individual, and black lines representing the variation in RMR for each individual. [file peerj-12-18168-s007.pdf]
